# Ultrasonic-Assisted Extraction and Gastrointestinal Digestion Characteristics of Polysaccharides Extracted from *Mallotus oblongfolius*

**DOI:** 10.3390/foods13121799

**Published:** 2024-06-07

**Authors:** Gansheng Tan, Zhouwei Duan, Guanghua Xia, Tian Xin, Ling Yang, Feng Liu, Hui Xie

**Affiliations:** 1 Institute of Agro-Products Processing and Design, Hainan Academy of Agricultural Science, Haikou 571100, China; 15736623006@163.com (G.T.); universeduan@163.com (Z.D.); xt98726@163.com (T.X.); yangling0911@126.com (L.Y.); liufeng1257585207@163.com (F.L.); 2 College of Food Science and Technology, Hainan University, Haikou 570228, China; xiaguanghua2011@126.com; 3 Sanya Institute, Hainan Academy of Agricultural Sciences, Sanya 572000, China

**Keywords:** *Mallotus oblongfolius* polysaccharides (MOPS), ultrasonic extraction, gastrointestinal digestion, antioxidant properties, hypoglycaemia

## Abstract

The polysaccharides were extracted from the leaves of *Mallotus oblongifolius* (MO) using an ultrasonic-assisted extraction method in this study. The main variables affecting the yield of polysaccharides extracted from *Mallotus appallatus* (MOPS) were identified and optimized while concurrently investigating its antioxidant capacity, hypoglycemic activity, and digestive properties. The results indicated that the optimal ultrasound-assisted extraction of MOPS involved an ultrasound power of 200 W, a liquid-to-solid ratio of 25:1 (mL:g), an extraction temperature of 75 °C, and an ultrasound time of 45 min, leading to an extraction yield of (7.36 ± 0.45)% (*m/m*). The MOPS extract exhibited significant scavenging activity against DPPH and ABTS radicals with IC_50_ values of (25.65 ± 0.53) μg/mL and (100.38 ± 0.38) μg/mL, respectively. Furthermore, it effectively inhibited the enzymatic activities of α-glucosidase and α-amylase with IC_50_ values of (2.27 ± 0.07) mg/mL and (0.57 ± 0.04) mg/mL, respectively. The content of MOPS remained relatively stable in the stomach and small intestine; however, their ability to scavenge DPPH radicals and ABTS radicals and exhibit reducing power was attenuated, and the inhibition of α-amylase and α-glucosidase activity was diminished. In conclusion, the ultrasonic extraction of MOPS showed feasibility and revealed antioxidant and hypoglycemic effects. However, the activities were significantly reduced after gastric and small intestinal digestion despite no significant change in the MOPS content.

## 1. Introduction

Mallotus oblongfolius (MO) [*Mallotus oblongifolius* (Miq.) MuellArg], Euphorbiaceae, commonly known as mountain bitter tea, Maocha, etc. [1], is mainly distributed in Sumatra, the Sino–Indian Peninsula, and China’s Hainan region [2]. It is abundant in polyphenols, polysaccharides, β-sitosterol, volatile oils, and other bioactive compounds [3]. MO exhibits remarkable properties, including potent antioxidant, anti-aging, sialagogic, and anti-inflammatory effects [4]. Moreover, MO is frequently employed as a tea substitute [5]. However, the exploitation of its resources remains underutilized. Therefore, it is imperative to explore strategies for maximizing the high-value utilization of MO resources.

In recent years, the focus of MO research has predominantly centered on the analysis of chemical composition and evaluation of polyphenolic activity [6]. For example, the polyphenol extracted from MO has been demonstrated to possess robust antioxidant and antibacterial properties [7], as well as the ability to prevent ethanol damage to the gastric mucosa [8]. However, there have been no documented studies investigating MOPS. Plant polysaccharides have various activities, such as antioxidant [9,10], anti-obesity [11] and hypoglycaemic effects [12]. Consequently, they are widely used in the fields of functional food and medicine. Polysaccharides are the main active bioactive compounds in MO, and exploring the nutritional and functional properties of MOPS provides a crucial avenue for the high-value utilization of MO resources.

Plant polysaccharides can be extracted using various methods, such as hot water immersion, ultrasonic-assisted extraction [13], microwave-assisted extraction [14], and enzyme-assisted extraction [15]. The ultrasonic-assisted extraction method is particularly advantageous in terms of its operational simplicity and high efficiency in extracting the target compound [16]. The structure, content, antioxidant, and hypoglycemic properties of polysaccharides may exhibit variables in a variety of digestive tract enzymes and an acid-base environment, causing inconsistent patterns of change in gastrointestinal digestion. For example, Oolong tea polysaccharides can be slightly digested by the gastrointestinal tract [17], and Qingchengliu polysaccharides exhibit negligible effects during simulated gastrointestinal digestion [18]. Therefore, it is crucial to investigate the extraction process and gastrointestinal digestion characteristics of MOPS, which are applied in the fields of functional foods and pharmaceuticals, and thereby enhance the applicability and value proposition of MO. In this work, the ultrasonic-assisted extraction method was employed to extract MOPS from MO leaves while simultaneously identifying and optimizing the key variables influencing the yield of polysaccharides extracted from MOPS. Additionally, this study investigated the antioxidant capacity, hypoglycemic activity, and digestive properties of MOPS in order to enhance our understanding of its physiological activities and provide support for its high-value utilization.

## 2. Materials and Methods

### 2.1. Materials and Chemicals

The samples of *Mallotus oblongfolius* (MO) were collected in Tonggu Ling, Wenchang City, Hainan Province, in November 2022. The green fresh leaves were selected as the experimental sample. Subsequently, they were subjected to a drying process at 55 °C and subsequently crushed through a 40-mesh sieve to obtain the MO powder, which was utilized for crude polysaccharide extraction.

Glucose standard, activated carbon (Xilong Science Co., Ltd., Shanghai, China). Concentrated sulfuric acid, phenol, and hydrochloric acid (Sinopharm Chemical Reagent Co., Ltd., Beijing, China). Bovine serum albumin V, (3000 U/g), and pancreatin (4000 U/g) (Sigma-Aldrich, Saint Louis, MO, USA). Inulin (Aladdin Co. Ltd., Shanghai, China). All other reagents were of analytical grade.

### 2.2. Extraction of MOPS

MO powder (10 g) was added to distilled water (the liquid-to-solid ratio ranged from 10:1 to 30:1 mL/g), followed by ultrasonication (X0-5200DTS Ultrasonic Cleaning Machine Nanjing Xian’ou Instrument Manufacturing Co., Ltd., Nanjing, China). The extraction temperature ranged from 35 °C to 75 °C, while the extraction time varied between 15 and 75 min). The samples were centrifuged using a high-efficiency refrigerated centrifuge (Avanti JXN-30, Shanghai Hengfei Biotechnology Co., Shanghai, China) at 2000 r/min for 6 min to obtain the polysaccharide extract of Partridge tea. The extraction steps were repeated twice, and the extracts were combined. The crude polysaccharide extract of partridge tea was concentrated using vacuum rotary evaporation (CF312L-B Cooling Water Circulation Unit, RE212 Rotary Evaporator Japan Yamato Joon & Shanghai Co., Ltd., Shanghai, China) at 40 °C, followed by the addition of anhydrous ethanol in a volume four times that of the extract. The resulting mixture was allowed to stand at a constant temperature of 4 °C for 12 h. After that, the mixture was centrifuged at 4500 r/min for 15 min to collect the precipitate. A small quantity of water was added to dissolve the precipitate, and the Sevage method was employed for protein removal (using a 4:1 ratio of polysaccharide solution to Sevage reagent (chloroform/n-butanol volume ratio of 4:1 for the mixture of solutions)) [19]. The mixture was placed in a dispensing funnel, vigorously shaken for 15 min, and allowed to rest for 30 min, and then the protein layer and organic solvent layer were discarded. This process was repeated until no protein remained. Decolorization and concentration were performed using 4% activated carbon (ZiRBUS Freeze Dryer Shanghai Jipu Electronic Technology Co., Ltd., Shanghai, China). The resulting concentrate was collected and subjected to freeze-drying to obtain crude MOPS.

### 2.3. Calculation of MOPS Yield

#### 2.3.1. Drawing of Standard Curve 

Glucose content was determined by the phenol–sulfuric acid method [20] using spectrophotometer analysis (TU-1810 UV-visible Spectrophotometer Beijing Puyi General Instrument Co., Ltd., Beijing, China). The 0.01, 0.02, 0.03, 0.04, and 0.05 mg/mL of glucose standard solution were individually pipetted into separate vials with a 2 mL volume each. Subsequently, a 1 mL volume of a phenol solution containing a concentration of 6% was added to each vial, followed by vigorous shaking. The mixture was subjected to the addition of 5 mL of concentrated sulfuric acid in a vertical manner, followed by allowing it to stand for 5 min. Subsequently, the absorbance value at 490 nm was measured after immersing the sample in a water bath at 50 °C for 20 min. The regression equation for the glucose standard was established using distilled water as a blank control, with the mass concentration C (mg/mL) of glucose as the horizontal coordinate and the absorbance value A as the vertical coordinate: A = 14.226 C − 0.0163, *R*^2^ = 0.9956.

#### 2.3.2. Determination of MOPS Content

The MOPS content was determined using the phenol–sulfuric acid method, which is consistent with the methodology described in Section 2.3.1. The polysaccharide yield was calculated using the provided equation as follows:

$$MOPS\;yield\;(\%)=\frac{(A+0.0163)DV}{14.226m}\times 100$$

Herein, A is the concentration of the MOPS solution, mg/mL^−1^; D is the MOPS dilution; V is the volume of MOPS extract, mL; and m is the mass of MO powder, mg.

### 2.4. Optimization of MOPS Extraction Process

#### 2.4.1. Single Factor Experimental Design

The extraction conditions for 5 g of MO powder were determined based on the polysaccharide extraction rate. These conditions included an ultrasonic power of 200 W, a temperature of 55 °C, a liquid-to-solid ratio of 1:20 (g/mL), and a time of 30 min. The remaining conditions were held constant, and the impacts of ultrasonic power (100, 150, 200, 250, and 300 W), temperature (35, 45, 55, 65, and 75 °C), liquid-to-solid ratio (1:10, 1:15, 1:20, 1:25, and 1:30 g/mL), and time (15, 30, 45, 60, and 75 min) on the yield of MOPS were investigated in sequence. The effects of ultrasonic power (100, 150, 200, 250, 300 W), temperature (35, 45, 55, 65, and 75 °C), liquid-to-solid ratio (1:10, 1:15, 1:20, 1:25, and 1:30 g/mL), and time (15, 30, 45, 60, and 75 min) on the extraction rate of polysaccharide was investigated to obtain the optimal conditions for each factor.

#### 2.4.2. Response Surface Experimental Design

The polysaccharide dissolution approached equilibrium with a liquid–solid ratio of 20:1 (mL/g) while maintaining a relatively stable yield. Based on the results of the single-factor test, three factors (ultrasonic power, extraction temperature, and extraction time) that exerted a significant influence on the alteration in polysaccharide yield were selected as the experimental variables. The MOPS yield was utilized as the response variable for optimizing the extraction process of polysaccharides. The extraction process was optimized using the Box-Benhnken Design principle in Design Expert 13 software, employing a three-factor, three-level response surface test. The factors and levels of the central combination design are shown in Table 1.

### 2.5. Determination of Antioxidant Activity of MOPS

MOPS was formulated into different mass concentration gradients, and their antioxidant activities were determined by the DPPH [21] method, ABTS [22] method, and reducing power method [23].

### 2.6. Determination of Hypoglycaemic Activity of MOPS

The inhibitory effects of MOPS on α-glucosidase and α-amylase were determined to evaluate their hypoglycaemic activities [24].

### 2.7. The MOPS Experiment Simulates In Vitro Digestion

#### 2.7.1. Vitro Simulation of Oral Digestion

The simulated digestion method employed in this study was based on previous research with slight modifications [25]. In total, 15 mL of the sample solution with 15 mL of oral electrolytes (0.7644 g NaCl, 1.491 g KCl, 0.1332 g CaCl_2_ dissolved in 1000 mL of distilled water, pH adjusted to 6.9 ± 0.05 with 1 mol/L HCl and 1 mol/L NaHCO_3_) was taken. Additionally, 2.3 mg of α-amylase was added to the mixture, which was thoroughly mixed and then oscillated at a constant temperature of 37 °C for a duration of 2 h. At specific time intervals (0, 30, 90, 120, and 150 min), the remaining oral digest was extracted. Subsequently, a volume of 2 mL from each time point was extracted and subjected to enzyme inactivation by heating at 100 °C for 10 min before storage for subsequent analysis.

#### 2.7.2. Vitro Simulation of Gastric Digestion

The oral digestive fluid (20 mL) was combined with gastric electrolytes (0.22 g KCl, 0.12 g NaHCO_3_, 0.05 g CaCl_2_, 0.62 g NaCl dissolved in 200 mL of distilled water). The resulting mixture’s pH was adjusted to 2 using a solution of 1 M HCl. Subsequently, pepsin (27 mg) was thoroughly mixed into the solution and subjected to oscillation at a constant temperature of 37 °C for a duration of 2 h. In total, 2 mL of gastric digestive fluid was removed from the stomach at 0, 30, 90, and 120 min, and 2 mL of gastric digestive fluid was inactivated at 100 °C for 10 min and stored for further digestion experiments. After 150 min, an additional two milliliters were withdrawn for testing while the enzyme was again inactivated at 100 °C for 10 min. The remaining gastric digest was utilized for further digestion experiments.

### 2.8. Vitro Simulation of Enteral Digestion

The 20 mL of gastric digestive fluid and 20 mL of intestinal electrolytes (0.065 g KCl, 0.54 g NaCl, 0.033 g CaCl_2_ were dissolved in 100 mL of distilled water. The pH of the mixture was adjusted to 7 (using 1 mol/L NaHCO_3_) and was thoroughly mixed. Subsequently, a solution containing 160 mg of bile salts and 200 mg of pancreatic enzymes was vigorously shaken at a constant temperature of 37 °C for 2 h. The sample was then incubated at temperatures of 0, 30, 90, and 120 °C for a duration of 2 h each. At time intervals of 0, 30, 90, and 150 min during the incubation period, aliquots of the solution (2 mL) were withdrawn. Following this step, the enzyme was inactivated by heating at a temperature of 100 °C for10 min before being stored for further testing.

### 2.9. Statistical Analysis

Each experiment was repeated three times, and the data quality was assessed by calculating the standard deviation (*SD*). The experimental design was conducted using Design Expert 13 software, while graphing was performed using Origin 8.5 software. The data were analyzed for statistical significance using IBM SPSS Statistics 23 software through one-way ANOVA. A * *p* < 0.05 was considered indicative of a significant difference, while a ** *p* < 0.01 indicated a highly significant difference.

## 3. Results and Analysis

### 3.1. Results of the Single-Factor Test

**Figure 1:**
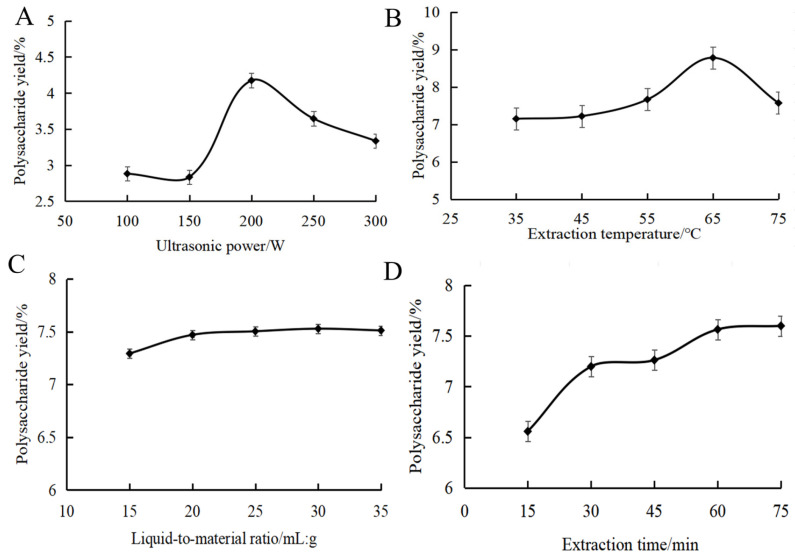
Effect of ultrasonic power (**A**), extraction temperature (**B**), liquid-to-material ratio (**C**) and extraction time (**D**) on the polysaccharide yield of partridge tea.

Figure 1A shows the effect of ultrasonic power on the yield of MOPS. The yield of MOPS exhibited an initial increase followed by a subsequent decrease in response to the varying ultrasonic power, ultimately reaching its peak value at 4.17 ± 0.02% when the ultrasonic power was set at 200 W. This might be because cavitation was enhanced with the increase in ultrasonic power. The mechanical vibration effect of ultrasound shattered the cell wall, releasing more polysaccharides and thereby increasing the MOPS yield. When the ultrasonic power exceeded 200 W, the intense ultrasonic action promoted the cleavage of polysaccharide molecular chains, causing a decrease in the yield [26]. It was appropriate to choose ultrasonic power at around 200 W.

The effect of extraction temperature on the yield of MOPS is shown in Figure 1B. As can be seen from the figure, an increase in ultrasonication time led to a rise in MOPS yield followed by a slight decrease, with the maximum polysaccharide yield achieved at 60 min (7.52 ± 0.05%). Subsequently, as the temperature continued to rise, there was a decrease in polysaccharide yield. This phenomenon can be attributed to the intensified diffusion of polysaccharide molecules and subsequent increase in solubility at temperatures above 65 °C. Excessively high temperatures above 65 °C led to the cleavage of polysaccharides, resulting in a decrease in polysaccharide yield [27]. It was appropriate to choose the extraction temperature at around 65 °C.

The effect of the liquid-to-material ratio on the yield of MOPS can be seen in Figure 1C. The MOPS yield initially increased and then reached a plateau as the liquid ratio increased. Due to increased solvent volume, the polysaccharides dissolved sufficiently at the liquid ratio below 20:1 mL/g, resulting in an enhanced yield. However, the dissolution of polysaccharides reached an equilibrium at the liquid ratio of more than 20:1 mL/g; further increasing the liquid ratio did not have a significant effect on the solubilization of polysaccharides [28]. Therefore, the liquid–liquid ratio was selected as 20:1 mL/g in this study.

The effect of the ultrasound time on the yield of MOPS can be seen in Figure 1D. As depicted, an increase in ultrasonication time led to a rise in MOPS yield followed by a slight decrease, with the maximum polysaccharide yield achieved at 60 min (7.52 ± 0.05%). This phenomenon may be attributed to enhanced cell fragmentation and the dissociation of polysaccharides, resulting in an increased yield within the first 60 min of ultrasound exposure. However, when the ultrasonic treatment time was extended beyond 60 min, the polysaccharide structure may change due to the increase in temperature, resulting in a decrease in the yield of polysaccharides [29]. Therefore, it was deemed appropriate to choose an approximate 60 min duration for the sonication.

### 3.2. Response Surface Optimization of MOPS Extraction Experiment

#### 3.2.1. Regression Equations

The data in Table 2 were analyzed using the Box-Bohnken method, with the MOPS yield being considered as the response variable. The regression equation of the MOPS yield Y on the coded values of ultrasonic power (A), temperature (B), and time (C) was obtained as Y = 6.64 – 0.4715A − 0.0199B − 0.0126C + 0.0464AB + 0.0453AC + 0.1419BC + 0.2938A^2^ – 0.3437B^2^ – 0.2754C^2^. The results of the multiple regression equation analysis of the factors influencing the MOPS yield are presented in Table 3. From the table, it is evident that the model exhibits a highly significant *p*-value (<0.0001) and a non-significant misfit term (*p* = 0.7566 > 0.05), indicating its ability to accurately reflect the experimental conditions. Furthermore, with a coefficient of variation of 0.91% (<10%), the model demonstrates excellent experimental stability and minimal susceptibility to external factors. The regression model yielded an R^2^ value of 0.9846 and an adjusted R^2^_Adj_ value of 0.9647, indicating a strong correlation between the experimental and actual models, with over 98% of the true values accurately predicted by the model. According to Table 3, BC exhibited a significant effect on the MOPS yield (*p* < 0.01). A, A^2^, B^2^, and C^2^ demonstrated highly significant effects on the MOPS yield (*p* < 0.01), while the remaining factors showed no significant effect. Therefore, the order of the three factors influencing the yield of MOPS was determined to be A > B > C, specifically ultrasonic power> extraction temperature > extraction time.

#### 3.2.2. Response Surface Analysis

**Figure 2:**
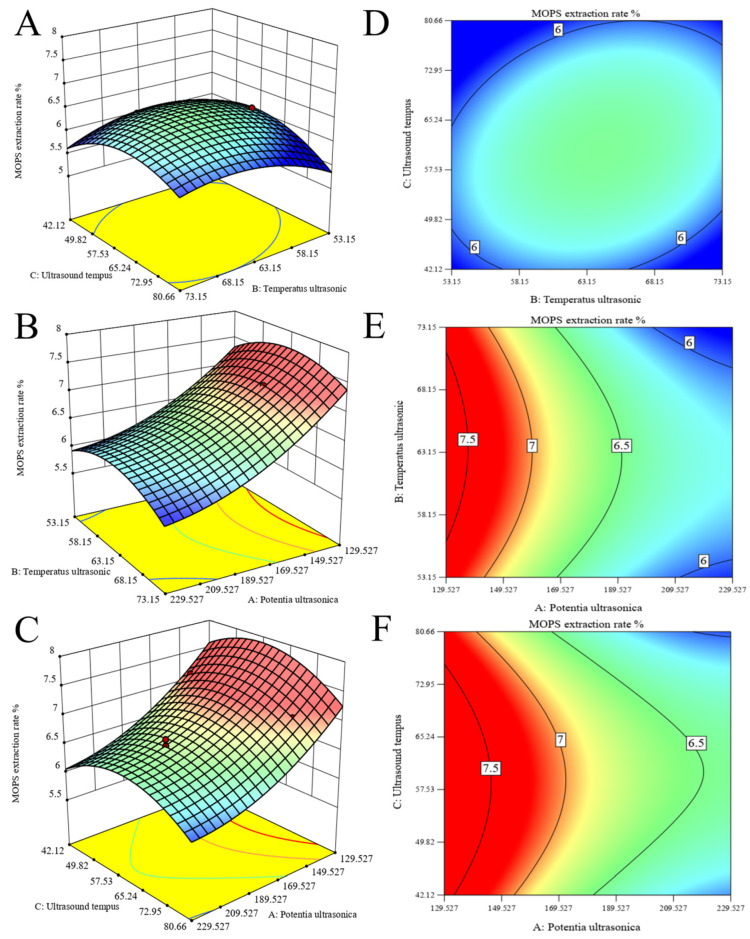
Optimization of MOPS extraction. Response surface plots (**A**–**C**) and contour lines (**D**–**F**) of ultrasonic extraction interactions were obtained, and extraction temperature, extraction time, and ultrasonic power were used as independent variables to optimize polysaccharide extraction.

The response surfaces and contour plots are effective tools for visualizing the extent of interaction influence on the values of the response surface [30]. As shown in Figure 2A,D, the relationship observed between extraction temperature and time demonstrates a steep slope and an elliptical contour line, indicating a significant interaction between these two factors. However, the surface of ultrasonic power and temperature exhibits a gentle slope and round contour lines, indicating a weak interaction between the two variables, as observed in Figure 2B,E. In addition, as depicted in Figure 2C,F, the gradients of the ultrasonic power–time surfaces exhibit a gentle inclination with circular contour lines, suggesting a weak interaction between these two variables.

#### 3.2.3. Optimized Process Validation Experiments

**Figure 3:**
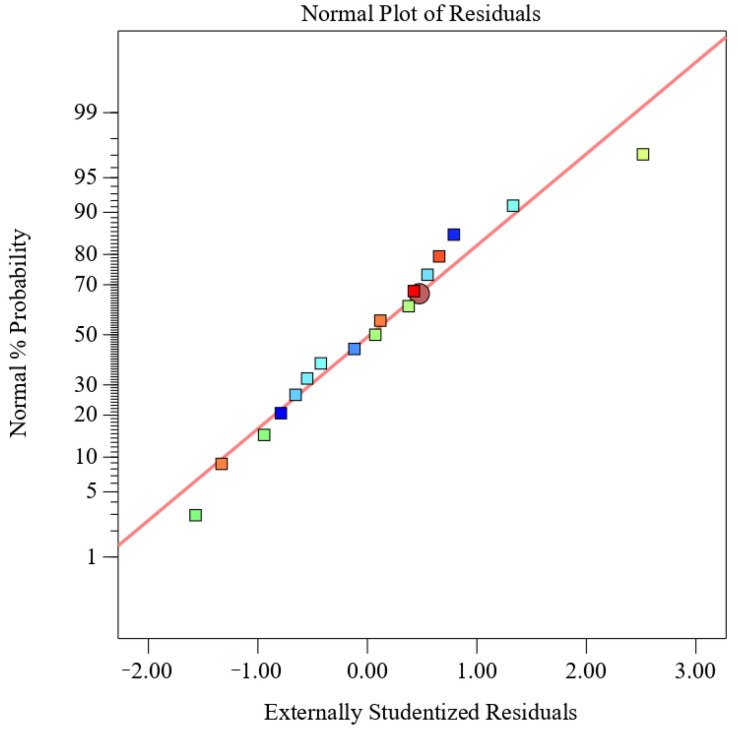
Normal probability plot of residuals.

From the normal probability of residuals in Figure 3, it can be seen that the normality assumption is supported by the straight-line approximation of the residual curves, indicating the feasibility of the proposed model for predicting MOPS yield. The optimal extraction conditions for achieving the highest yield of tea polysaccharide (7.22%) were determined using Design Expert 13 software, with a power input of 218 W, temperature set at 75 °C, and an extraction time of 45 min. According to the possibility and convenience of experimental operation, the extraction conditions were adjusted to 200 W, 75 °C, and 45 min. Three replicates were conducted under these adjusted conditions, resulting in an MOPS yield of (7.36 ± 0.45)%, which was consistent with the theoretical prediction. These findings demonstrate that it was feasible to extract MOPS using these conditions.

### 3.3. Antioxidant Effects of MOPS

#### 3.3.1. Free radical Scavenging and Reducing Capacity of MOPS

**Figure 4:**
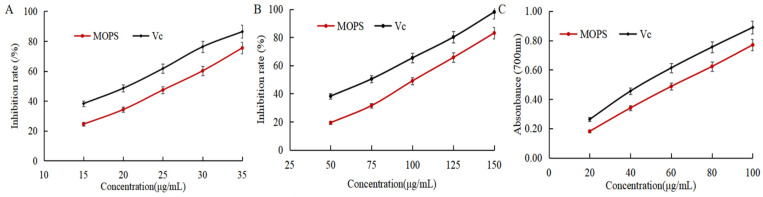
Effects of MOPS on the scavenging of DPPH free radicals (**A**), scavenging of ABTS free radicals (**B**), and reducing power (**C**).

DPPH, ABTS radical scavenging capacity, and reducing power is usually used to assess the antioxidant activity index [31]. As shown in Figure 4A,B, the scavenging rate of DPPH and ABTS free radicals gradually increased with the increase in the mass concentration of MOPS. The IC_50_ of DPPH and ABTS radical scavenging by MOPS were (25.65 ± 0.53) and (100.38 ± 0.38) μg/mL, respectively, which were higher than the IC_50_ (20.79 ± 0.32) and (72.41 ± 0.59) μg/mL of Vc, indicating that the MOPS had the scavenging ability of DPPH and ABTS free radicals, but the anti-free radical ability was lower than that of Vc. The reducing power of MOPS extract and VC increased with the increase in extract mass concentration, as depicted in Figure 4C. The reduction ability of MOPS can be indirectly reflected by the absorption value at 700 nm, and the greater the absorbance, the stronger the reduction ability [32]. When the concentrations of MOPS extract and VC were 60 μg/mL, the corresponding absorbance values were 0.613 ± 0.08 and 0.636 ± 0.11, respectively, indicating that the reducing power of the MOPS extract was comparatively weaker than that of Vc.

#### 3.3.2. Hypoglycaemic Effect of MOPS

**Figure 5:**
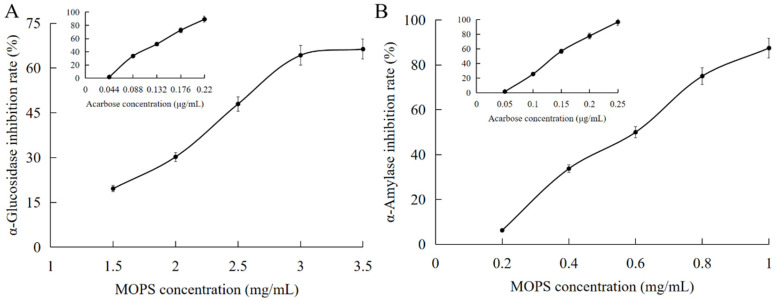
Hypoglycaemic effects of MOPS. Inhibition of α- glucosidase by MOPS (**A**) and α-amylase activity by MOPS (**B**).

The enzymes α-glucosidase and α-amylase play pivotal roles in regulating blood glucose levels in the human body, making them crucial determinants of both hyperglycemia and hypoglycemia. Consequently, they serve as vital indicators for evaluating the efficacy of hypoglycemic intervention [33]. The inhibitory effect of MOPS on α-amylase increased significantly from (6.25 ± 0.11)% to (87.5 ± 0.44)% as the mass concentration ranged from 0.2 mg/mL to 1 mg/mL, as illustrated in Figure 5A,B. The IC_50_ value for this inhibition was determined to be (0.57 ± 0.04) mg/mL, which is lower than that of acarbose with an IC_50_ value of (0.147 ± 0.07) μg/mL, indicating a comparatively weaker potency of MOPS in inhibiting α-amylase when compared to acarbose.

### 3.4. Simulation of In Vitro Digestion of MOPS

#### 3.4.1. Polysaccharide Content

As presented in Table 4, it demonstrates that there are no statistically significant differences (*p* > 0.05) observed in the MOPS content during the simulated oral, gastric, and intestinal digestion stages. Furthermore, no significant variations were found across different time points within these digestive environments, indicating that neither the oral, gastric, or intestinal conditions exerted a substantial impact on the release, or degradation of MOPS.

#### 3.4.2. Antioxidant Properties of MOPS

**Figure 6:**
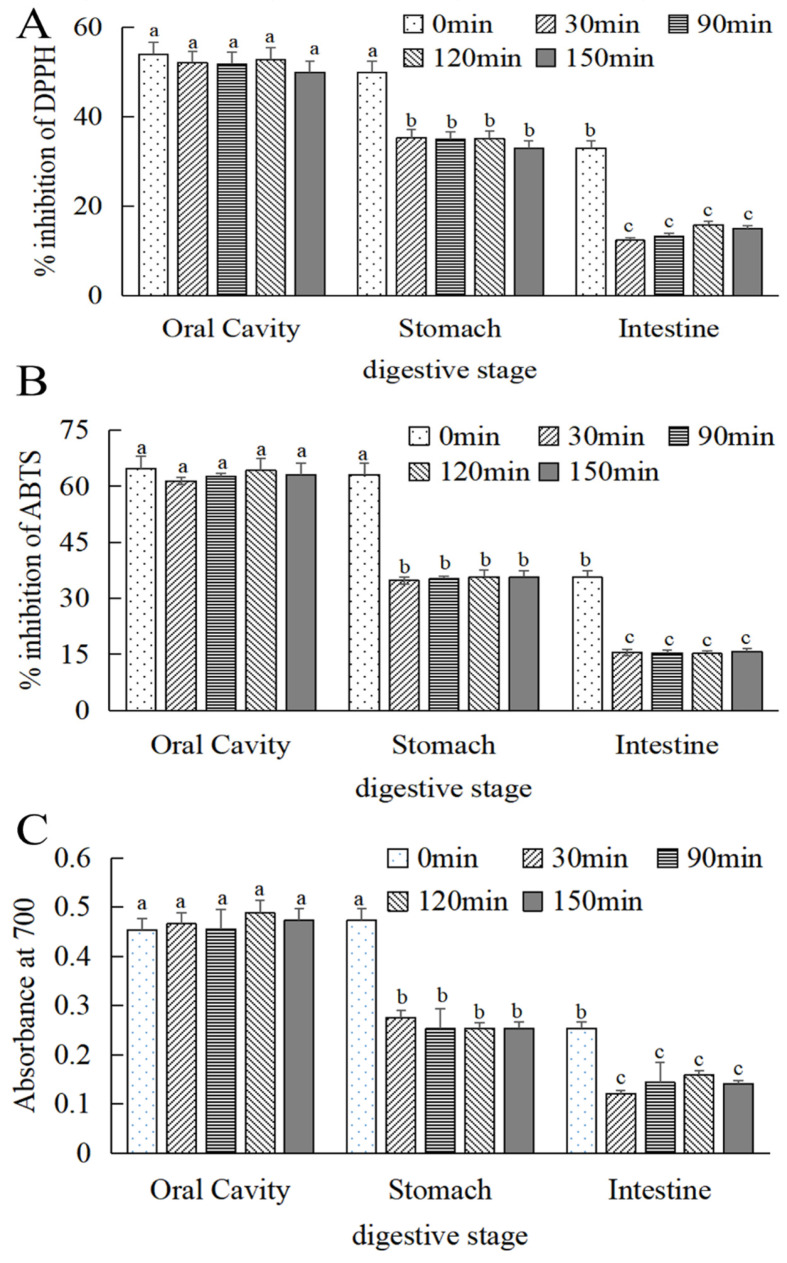
Changes in antioxidant activity of MOPS at different digestive sites. (**A**) DPPH radical scavenging activity, (**B**) ABTS radical scavenging activity, and (**C**) iron reduction capacity. Different lowercase letters indicate significant differences between different treatments (*p* < 0.05).

As can be seen in Figure 6A–C, the free radical scavenging effect and reduction in the power of MOPS on DPPH and ABTS were significantly reduced (*p* < 0.05) in simulated oral, gastric, and intestinal cavities. With the extension of digestion time, the free radical scavenging and reducing the energy of MOPS on DPPH and ABTS tended to be stable after digestion for 30 min. Compared to the simulated stomach and intestine at 0 min, the scavenging activity of DPPH decreased by (33.24 ± 0.24)% and (84.76 ± 0.87)%, respectively, after 30 min of gastric and intestinal digestion. Similarly, ABTS activity decreased by (43.55 ± 0.24)% and (75 ± 0.08)%, while the absorption value decreased by (34.6 ± 0.55)% and (52.36 ± 0.87)%, respectively.

#### 3.4.3. Hypoglycaemic Activity of MOPS

**Figure 7:**
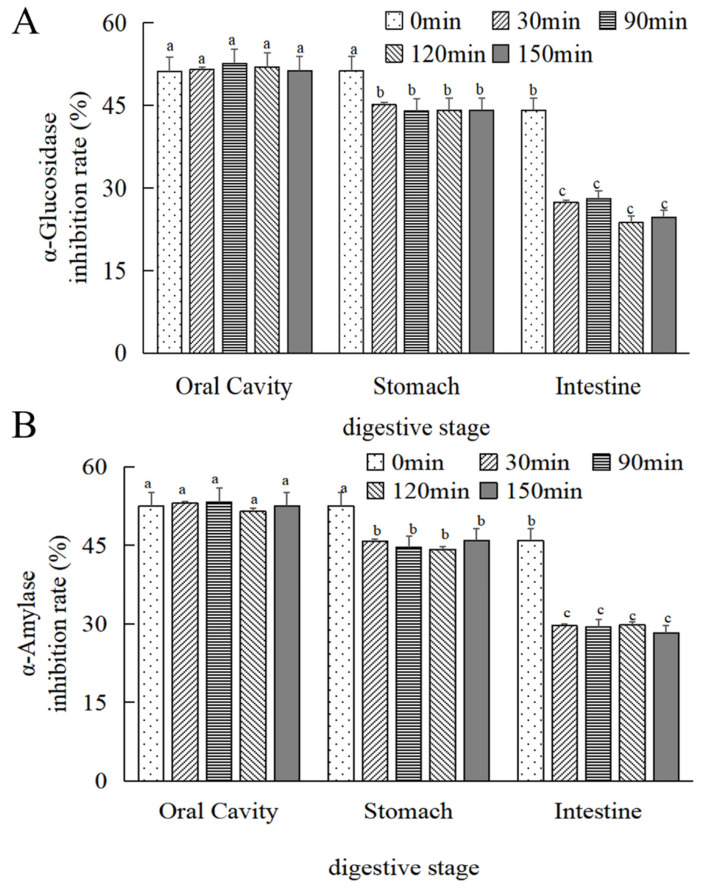
Changes in the hypoglycaemic activity of MOPS at different digestive sites. (**A**) α-glucosidase inhibitory activity and (**B**) α-amylase inhibitory activity. Compared with the 0 min simulated oral cavity. Different lowercase letters indicate significant differences between different treatments (*p* < 0.05).

As depicted in Figure 7A,B, there was no statistically significant difference (*p* > 0.05) observed in the alteration of the α-glucosidase inhibitory activity of MOPS before and after oral digestion with increasing digestion time. Following 30 min of simulated gastric and intestinal digestion, a reduction in α-glucosidase inhibitory activity by (12 ± 0.55)% and (37.86 ± 0.27)%, respectively, was observed compared to the initial 0 min simulation; similarly, α-amylase inhibitory activity decreased by (7.12 ± 0.24)% and (35.47 ± 0.38)%, respectively, and was stabilized at 30 min (*p* > 0.05).

## 4. Discussion

The objective of this study was to examine the extraction process of MOPS. The test variables, namely ultrasonic power, extraction temperature, and extraction time, were chosen based on a one-factor approach. The yield of partridge tea polysaccharide was used as the evaluation criterion of the extraction process. The extraction process conditions were effectively optimized using the response surface method. The optimal conditions for extracting MOPS were determined as follows: ultrasonic power of 200 W, liquid-to-material ratio of 1:25, extraction temperature of 75 °C, and extraction time of 45 min.

The antioxidant activity of polysaccharides and their derivatives are usually assessed in terms of free radical scavenging and reducing capacities. The antioxidant activity of polysaccharides is influenced by various factors, including molecular weight, conformation, type of glycosidic bond, and monosaccharide composition [34]. There is a higher free radical scavenging activity of polymers with highly branched and β-glycosidic bonds [35], as well as plant polysaccharides containing high amounts of galacturonic acid [36]. Additionally, the electrophilic ketone or aldehyde groups present in glycoaldehyde augment the activity of hydrogen atoms and facilitate their dissociation from hydroxyl bonds, thereby enhancing their antioxidant effects [37]. The present study demonstrated the alterations in the in vitro DPPH and ABTS radical scavenging activity and reduced the power of MOPS (Figure 4). The in vitro antioxidant capacity exhibited an upward trend with the increasing concentration of the extracts. The IC_50_ values were (25.65 ± 0.53) and (100.38 ± 0.38) μg/mL for the free radical scavenging rate of DPPH and ABTS, respectively. This is consistent with the antioxidant properties of polysaccharides from large bulbous mushrooms [38]. But the IC_50_ values were different, probably due to variations in the structures and compositions of the polysaccharides, which caused the difference in antioxidant capacity. Following in vitro simulated oral, gastric, and intestinal digestion, the total content of MOPS exhibited no significant change (*p* > 0.05). This might be attributed to the non-digestible nature of MOPS, rendering it ineffective for utilization by the stomach and small intestine. This is similar to the trend of changes in the polysaccharide content of green willow after in vitro digestion [39]. Moreover, the in vitro digestion of MOPS resulted in a weakened ability to scavenge DPPH and ABTS free radicals as well as their reduction. This finding is consistent with Zhu et al.’s study on alterations in the antioxidant activity of Lobelia polysaccharides during in vitro gastrointestinal digestion. These changes might be attributed to the acidic pH conditions during oral, gastric, and intestinal digestion, leading to a reduction in the glucuronic acid content of the polysaccharides. Additionally, the digestive enzymes and temperature could also contribute to modifications in the structure and properties, which, in turn, caused changes in the activity [40]. The phenolic acids present in MOPS exhibited instability under conditions of partial acidity or alkalinity, leading to their degradation and the formation of other compounds, thereby impacting their antioxidant activity [41,42,43].

α-glucosidase and α-amylase are hydrolases located on the surface of small intestinal epithelial cells, which catalyze the hydrolysis of α-glucose to glucose by cleaving the α-1, 4-glucoside bond of α-glucosidase and α-amylase [44]. The process described leads to an elevation in the body’s blood glucose levels. The competitive inhibition of α-glucosidase activity by enzyme inhibitors effectively delays or inhibits the hydrolysis of disaccharides and oligosaccharides into monosaccharides, thereby reducing or inhibiting glucose absorption by the body and subsequently lowering postprandial blood glucose levels [45].

Polysaccharides are the most common natural α-glucosidase inhibitors. The present study investigated the inhibitory effects of MOPS on the enzymatic activities of α-glucosidase and α-amylase. The IC50 values for α-glucosidase and α-amylase were (2.27 ± 0.07) mg/mL and (0.57 ± 0.04) mg/mL, respectively. The inhibition of α-glucosidase increased from (19.61 ± 0.15)% to (66.31 ± 0.22)%, and that of α-amylase increased from (6.25 ± 0.11)% to (87.5 ± 0.44)% with increasing MOPS concentration. These results are consistent with the changes in α-glucosidase and amylase activities in response to stilbene polysaccharide and buckwheat polysaccharide [44,46]. Furthermore, a positive correlation was observed between the inhibition rate and the concentration of polysaccharides. The hypoglycemic activities of MOPS were significantly diminished (*p* < 0.05) after simulating oral, gastric, and intestinal digestion in vitro. This finding is consistent with the observed trend in α-glucosidase inhibitory activity during simulated in vitro gastrointestinal digestion using Birch brown porcupine polysaccharide [47]. It might be that the composition and molecular weight distribution of MOPS changed after gastrointestinal digestion, consequently affecting their hypoglycemic potential [48]. In addition, there were differences in the interaction mode of α-glucosidase and amylase with the products of MOPS after simulated digestion through different stages of the oral, gastric, and intestinal tracts, and pectin-like polysaccharides containing galacturonic acid glycan regions and rhamnogalacturonic acid regions containing neutral sugar side chains were usually found to have hypoglycaemic activity [44]. Only a minimal amount of galacturonic acid was released during the oral, gastric, and intestinal digestion process [49,50,51]. The activity of α-glucosidase and amylase may be influenced by the gastrointestinal digestive environment, while the acidic conditions can impact the polysaccharide structure, leading to alterations in its functionality.

In summary, MOPS possesses the capacity to effectively scavenge DPPH radicals and ABTS radicals, as well as inhibiting the enzymatic activities of α-glucosidase and α-amylase. Notably, no significant alterations in the MOPS content were observed within the oral cavity, stomach, and small intestine. However, MOPS, by simulated in vitro digestion, reduced the scavenging potential of DPPH radicals and ABTS radicals, as well as the reducing power, and diminished the inhibitory effects of α-amylase and α-glucosidase.

The results demonstrate that the ultrasound-assisted method was suitable for extracting MOPS with potent antioxidant and hypoglycemic properties. There was no significant change in the content of MOPS after oral, gastric, and small intestinal digestion, and their antioxidant and hypoglycemic activities were significantly attenuated during gastrointestinal digestion.

## Figures and Tables

**Table 1:** Independent variables and their levels used in the response surface design.

Independent Variables	Levels		
	−1	0	1
A Ultrasonic power/W	150	200	250
B extraction temperature/°C	55	65	75
C extraction time/min	45	60	75

**Table 2:** Response surface test design and results.

Serial Number	A Ultrasonic Power W	B Extraction Temperature °C	C Extraction Time min	MOPS Yield %
1	200	75	45	5.91
2	150	65	45	7.21
3	200	65	60	6.78
4	200	65	60	6.57
5	200	65	60	6.54
6	150	75	60	7.04
7	150	65	75	7.10
8	250	65	45	6.13
9	150	55	60	7.04
10	250	65	75	6.20
11	250	55	60	6.24
12	250	75	60	6.05
13	200	55	75	5.85
14	200	65	60	6.64
15	200	65	60	6.67
16	200	55	45	6.17
17	200	75	75	6.16

**Table 3:** Variance analysis of regression model.

Source of Variance	Sum of Squares	d*f*	Mean Squares	*f*-Value	*p* > f	*p*
Modeling	3.03	9	0.3363	49.58	<0.0001	**
A	1.78	1	1.78	262.21	<0.0001	**
B	0.0032	1	0.0032	0.4667	0.5165	
C	0.0013	1	0.0013	0.1881	0.6775	
AB	0.0086	1	0.0086	1.27	0.2965	
AC	0.0082	1	0.0082	1.21	0.3077	
BC	0.0805	1	0.0805	11.87	0.0108	*
A^2^	0.3635	1	0.3635	53.59	0.0002	**
B^2^	0.4973	1	0.4973	73.33	<0.0001	**
C^2^	0.3193	1	0.3193	47.08	0.0002	**
Residual	0.0475	7	0.0068			
Lost proposal	0.0111	3	0.0037	0.4076	0.7566	
Pure error	0.0364	4	0.0091			
Total deviation	3.07	16				
R^2^	0.9846					
Adjusted R^2^	0.9647					
Predicted R^2^	0.9237					

Note: ** indicates a highly significant difference (*p* < 0.01) and * indicates a significant difference (0.01 < *p* < 0.05).

**Table 4:** Changes in the content of MOPS during digestion in the oral cavity, stomach and small intestine.

	Digestion Time/min	MOPS Content (×10^−3^ mg/mL)
oral cavity	0	157.26 ± 2.11
	30	153.42 ± 0.24
	90	156.87 ± 0.76
	120	154.81 ± 0.87
	150	155.69 ± 0.62
gastric	0	152.76 ± 0.32
	30	156.72 ± 0.23
	90	154.17 ± 0.32
	120	157.83 ± 0.65
	150	161.85 ± 1.77
colorectal	0	163.33 ± 2.32
	30	164.85 ± 0.78
	90	161.61 ± 0.63
	120	162.32 ± 0.99
	150	160.45 ± 1.20

## Data Availability

The original contributions presented in the study are included in the article, further inquiries can be directed to the corresponding author.
